# Early protein delivery in critically ill patients with acute kidney injury: *post hoc* analysis of a multicenter cluster-randomized controlled trial

**DOI:** 10.1093/burnst/tkae027

**Published:** 2024-07-24

**Authors:** Cheng Lv, Lingliang Zhou, Yufeng Zhou, Charles Chin Han Lew, Zheng-Yii Lee, M Shahnaz Hasan, Baiqiang Li, Yang Liu, Jiajia Lin, Wenjian Mao, Christian Stoppe, Arthur Raymond Hubert van Zanten, Weiqin Li, Yuxiu Liu, Lu Ke, the Chinese Critical Care Nutrition Trials Group (CCCNTG), Cheng Lv, Lingliang Zhou, Yufeng Zhou, Charles Chin Han Lew, Zheng-Yii Lee, M Shahnaz Hasan, Baiqiang Li, Yang Liu, Jiajia Lin, Wenjian Mao, Christian Stoppe, Arthur Raymond Hubert van Zanten, Weiqin Li, Yuxiu Liu, Lu Ke

**Affiliations:** Department of Critical Care Medicine, Jinling Hospital, Affiliated Hospital of Medical School, Nanjing University, 22 Hankou Road, Gulou District, Nanjing 210093, China; Department of Critical Care Medicine, Jinling Hospital, Affiliated Hospital of Medical School, Southeast University, 87 Ding Jiaqiao, Gulou District, Nanjing 210009, China; Department of Biostatistics, School of Public Health, Southern Medical University, 1023-1063 Shatai South Road, Baiyun District, Guangzhou 510515, China; Department of Dietetics and Nutrition, Ng Teng Fong General Hospital, Singapore, Singapore 1 Jurong East Street 21, Singapore; Department of Cardiac Anesthesiology and Intensive Care Medicine, Charité Berlin, Charitéplatz 1, 10117 Berlin, Germany; Department of Anaesthesiology, Faculty of Medicine, University of Malaya, Lembah Pantai, Kuala Lumpur 50603, Malaysia; Department of Anaesthesiology, Faculty of Medicine, University of Malaya, Lembah Pantai, Kuala Lumpur 50603, Malaysia; Department of Anaesthesiology, Universiti Malaya Medical Centre, Lembah Pantai, Kuala Lumpur 59100, Malaysia; Department of Critical Care Medicine, Jinling Hospital, Affiliated Hospital of Medical School, Nanjing University, 22 Hankou Road, Gulou District, Nanjing 210093, China; National Institute of Healthcare Data Science, Nanjing University, 22 Hankou Road, Gulou District, Nanjing 210093, China; Research Institute of Critical Care Medicine and Emergency Rescue At Nanjing University, 22 Hankou Road, Gulou District, Nanjing 210093, Jiangsu Province, China; Department of Critical Care Medicine, Jinling Hospital, Affiliated Hospital of Medical School, Southeast University, 87 Ding Jiaqiao, Gulou District, Nanjing 210009, China; Department of Critical Care Medicine, Jinling Hospital, Affiliated Hospital of Medical School, Nanjing University, 22 Hankou Road, Gulou District, Nanjing 210093, China; Department of Critical Care Medicine, Jinling Hospital, Affiliated Hospital of Medical School, Nanjing University, 22 Hankou Road, Gulou District, Nanjing 210093, China; Department of Cardiac Anesthesiology and Intensive Care Medicine, Charité Berlin, Charitéplatz 1, 10117 Berlin, Germany; Department of Anaesthesiology, Intensive Care, Emergency and Pain Medicine, University Hospital Würzburg, Oberdürrbacher Str. 6, 97080, Würzburg, Germany; Department of Intensive Care, Gelderse Vallei Hospital, Willy Brandtlaan 10, 6716 RP Ede, The Netherlands; Division of Human Nutrition and Health, Wageningen University & Research, Helix (Building 124), Stippeneng 4, 6708 WE Wageningen, The Netherlands; Department of Critical Care Medicine, Jinling Hospital, Affiliated Hospital of Medical School, Nanjing University, 22 Hankou Road, Gulou District, Nanjing 210093, China; National Institute of Healthcare Data Science, Nanjing University, 22 Hankou Road, Gulou District, Nanjing 210093, China; Research Institute of Critical Care Medicine and Emergency Rescue At Nanjing University, 22 Hankou Road, Gulou District, Nanjing 210093, Jiangsu Province, China; Department of Biostatistics, School of Public Health, Southern Medical University, 1023-1063 Shatai South Road, Baiyun District, Guangzhou 510515, China; National Institute of Healthcare Data Science, Nanjing University, 22 Hankou Road, Gulou District, Nanjing 210093, China; Research Institute of Critical Care Medicine and Emergency Rescue At Nanjing University, 22 Hankou Road, Gulou District, Nanjing 210093, Jiangsu Province, China; Department of Critical Care Medicine, Jinling Hospital, Affiliated Hospital of Medical School, Nanjing University, 22 Hankou Road, Gulou District, Nanjing 210093, China; National Institute of Healthcare Data Science, Nanjing University, 22 Hankou Road, Gulou District, Nanjing 210093, China; Research Institute of Critical Care Medicine and Emergency Rescue At Nanjing University, 22 Hankou Road, Gulou District, Nanjing 210093, Jiangsu Province, China

**Keywords:** Protein delivery, Mortality, Acute kidney injury, Renal replacement therapy, Critical illness

## Abstract

**Background:**

There is controversy over the optimal early protein delivery in critically ill patients with acute kidney injury (AKI). This study aims to evaluate whether the association between early protein delivery and 28-day mortality was impacted by the presence of AKI in critically ill patients.

**Methods:**

This is a *post hoc* analysis of data from a multicenter cluster-randomised controlled trial enrolling newly admitted critically ill patients (n = 2772). Participants without chronic kidney disease and with complete data concerning baseline renal function were included in this study. The primary outcome was 28-day mortality. Cox proportional hazards models were used to analyze the association between early protein delivery, reflected by mean protein delivery from day 3–5 after enrollment, 28-day mortality and whether baseline AKI stages interacted with this association.

**Results:**

Overall, 2552 patients were included, among whom 567 (22.2%) had AKI at enrollment (111 stage I, 87 stage II, 369 stage III). Mean early protein delivery was 0.60 ± 0.38 g/kg/day among the study patients. In the overall study cohort, each 0.1 g/kg/day increase in protein delivery was associated with a 5% reduction in 28-day mortality[hazard ratio (HR) = 0.95; 95% confidence interval (CI) 0.92–0.98, *p* < 0.001]. The association between early protein delivery and 28-day mortality significantly interacted with baseline AKI stages (adjusted interaction *p* = 0.028). Each 0.1 g/kg/day increase in early protein delivery was associated with a 4% reduction in 28-day mortality (HR = 0.96; 95%CI 0.92–0.99, *p* = 0.011) among patients without AKI and 9% (HR = 0.91; 95%CI 0.84–0.99, *p* = 0.021) among those with AKI stage III. However, such associations cannot be observed among patients with AKI stages I and II.

**Conclusions:**

Increased early protein delivery (up to close to the guideline recommendation) was associated with reduced 28-day mortality in critically ill patients without AKI and with AKI stage III, but not in those with AKI stage I or II.

Highlights

- By analyzing data from a multicenter cluster-randomised controlled trial (n = 2772), we provide insights into optimal early protein delivery in critically ill patients with various AKI stages.
- Overall, in critically ill patients, increased early protein delivery (up to close to the guideline recommendation) was significantly associated with a reduction in 28-day mortality.
- Early protein delivery and 28-day mortality significantly interacted with baseline AKI stages. Increased early protein delivery was only associated with reduced 28-day mortality in patients without AKI and with AKI stage III, but not in those with AKI stage I or II.
- More high-quality evidence is needed to evaluate the role of renal function in protein delivery in critically ill patients.

## Background

Severe protein catabolism and massive muscle loss (nearly 2% of skeletal muscle per day on average [1]) are common during the early phase of critical illness [2,3]. The 2016 Society of Critical Care Medicine (SCCM)/American Society for Parenteral and Enteral Nutrition (ASPEN) guidelines and the 2018 European Society for Clinical Nutrition and Metabolism (ESPEN) guidelines recommend initiating early protein supplementation therapy within 24–48 h and reaching the target progressively within the acute phase of critical illness [4,5]. However, current nutrition guidelines lack specific recommendations for critically ill patients with acute kidney injury (AKI) [6].

From a physiological viewpoint, protein delivery could increase renal perfusion [7,8], which in turn improves glomerular filtration rate and urine output [9]. On the other hand, increased ureagenesis coupled with impaired muscle protein synthesis might be a metabolic burden due to excessive protein breakdown [10,11]. In a recent large trial, a high-protein strategy was associated with worse outcomes for patients with AKI in a subgroup analysis [12]. So far, the optimal early protein delivery strategy in these patients remains controversial [13–17]. Moreover, specific stages of AKI may impact the effect of protein delivery on clinical outcomes, which has been rarely studied in the literature.

To investigate whether baseline AKI stages interact with the associations between early protein delivery and clinical outcomes in critically ill patients, we performed a *post hoc* analysis using data from a large multicenter cluster-randomized trial.

## Methods

### Study design and patients

This study is a *post hoc* analysis of data from the multicenter, cluster-randomised controlled NEED trial [18]. The NEED trial assessed the effect of actively implementing an evidence-based feeding guideline on clinical outcomes in critically ill patients. A total of 2772 patients from 90 intensive care units (ICUs) across China were enrolled between 26 March 2018 and 4 July 2019. The trial was approved by the ethics committee of Jinling Hospital (22017NZKY-019-02) and registered with the ISRCTN registry (ISRCTN12233792). Data storage and academic usage of de-identified data after the trial were covered in the ethical approval. Informed consent was obtained from the patients or their next of kin before enrollment.

This *post hoc* analysis was performed in a subgroup of the study participants with complete data on 28-day mortality, baseline renal function and other baseline characteristics. Patients with chronic kidney disease upon enrollment, according to their admission records, were excluded. The complete eligibility criteria and full details on data collection of the NEED trial can be found in [18]. This report follows the Strengthening the Reporting of Observational Studies in Epidemiology (STROBE) reporting guideline for observational studies [19].

### Protein delivery and AKI

Early protein delivery was reflected by the mean protein delivery from trial days 3–5 [4]. Protein intake from enteral nutrition (EN), parenteral nutrition (PN) and/or protein supplements was included in the calculation of total protein delivery.

Baseline AKI was defined using Kidney Disease Improving Global Outcomes (KDIGO) classification: stage I is at least 26–52 μmol/l increase in serum creatinine from baseline within 48 h or 1.5–1.9-times baseline within 7 days; stage II is 2.0–2.9-times baseline within 7 days; and stage III is ≥3 times baseline within 7 days or increase to at least 353.6 μmol/l with an acute increase of >44.2 μmol/l, or undergoing renal replacement therapy (RRT) [20]. Since pre-acute illness creatinine values were not recorded in the original trial, the upper limit of normal (90 μmol/l for females and 110 μmol/l for males) was used as baseline creatinine.

### Study outcomes and data collection

The primary outcome was 28-day mortality. All data required in this *post hoc* analysis were extracted from the electronic database of the NEED trial. The baseline characteristics included age, gender, body mass index (BMI), acute gastrointestinal injury (AGI) score [21] and modified nutrition risk in critically ill (mNUTRIC) score [22], and disease severity scores, such as acute physiology and chronic health evaluation II (APACHE II) score [23] and sequential organ failure assessment (SOFA) score [24] at enrollment. Nutrition therapy variables included time to feed initiation, daily nutritional route, mean total daily protein and energy delivery, and feeding intolerance incidence. Feeding intolerance was reflected through three categories of common gastrointestinal symptoms defined in the feeding intolerance score [25], i.e. abdominal distension/pain, nausea/vomiting and diarrhea. Nutrition delivery was collected from days 3–5 upon enrollment or until discharge from the ICU or death, whichever occurred first.

### Statistical analysis

Statistical analyses were performed using R software version 4.2.2. After the Shapiro–Wilk test, continuous data are presented as mean ± standard deviation (SD) or median [interquartile range (IQR)] according to their normality. Categorical data are presented as numbers and percentages. Differences in baseline and nutritional characteristics among the four different categories of renal function were compared using the one-way analysis of variance (ANOVA) or the Kruskal–Wallis tests for continuous data and the chi-square test for categorical data. Kaplan–Meier survival curves depict mortality likelihood up to day 28.

Cox proportional hazards models were used to analyze the effect of early protein delivery on 28-day mortality. Candidate covariates with clinical significance were assessed for multi-correlation analysis, such as group allocation in the NEED trial, age, gender, BMI, AKI stage, AGI score, APACHE II score, SOFA score, mNUTRIC score and mean calorie delivery between days 3 and 5, and those without significant correlations were included in the multivariable models. AKI stage and AGI score were set as ordinal variables, whereas mNUTRIC and APACHE II scores were set as continuous variables. For the sensitivity analyses, Cox models were re-run with the day 3–5 range of mean early protein delivery extended to day 3–7 and with early protein delivery divided into three groups by tertiles.

To investigate the associations between protein delivery and 28-day mortality in patients with different baseline AKI stages, subgroup analyses were conducted. COX regression models were performed with the same adjustments as mentioned above. The fit of all the Cox models was assessed using Cox–Snell residuals. The proportional hazards assumption was tested by plotting Schoenfeld residuals. The trends of 28-day mortality with early protein delivery were modeled as restricted cubic splines with pre-specified knots [26]. A two-tailed *p*-value < 0.05 was considered significant.

## Results

### Patient population

Overall, 2552 patients were included in the current study (Figure S1, see online supplementary material). Of the study patients, 567 (22.2%) had AKI at enrollment (111 in stage I, 87 in stage II, 369 in stage III). Table 1 describes the baseline characteristics and clinical outcomes of the overall study population and each AKI subgroup. In general, most patients had mild gastrointestinal dysfunction (AGI grade I, n = 1813, 71%) with a relatively high nutritional risk (median mNUTRIC: 5, IQR: 3–6). The overall 28-day mortality was 14.9% (379/2552), and the median ICU-free days at 28 days was 7 (IQR: 0–17 days).

**Table 1:** Baseline characteristics and clinical outcomes in the study population

	**Total**	**Without AKI**	**AKI stage I**	**AKI stage II**	**AKI stage III**	***P*** **value**
Number (%)	2552	1985 (77.8)	111 (4.3)	87 (3.4)	369 (14.5)	
Age, years	62 [48, 74]	62 [49, 74]	64 [52.5, 79]	66 [49.5, 77.5]	58 [43,7]	<0.001
BMI (kg/m^2^)	22.86 [20.81, 24.57]	22.68 [20.76, 24.49]	22.58 [20.76, 24.97]	22.04 [20.88, 23.48]	23.14 [21.30, 25.25]	0.002
Gender (Male, %)	1716 (67.2)	1346 (67.8)	66 (59.5)	55 (63.2)	249 (67.5)	0.263
Group (intervention, %)	1268 (49.7)	1023 (51.5)	51 (45.9)	50 (57.5)	144 (39.0)	<0.001
APACHE II score	18 [14, 23]	17 [13, 22]	21 [16, 26]	24 [19.5, 28]	22 [16, 28]	<0.001
SOFA score	7 [5, 10]	7 [5, 9]	10 [7, 11.5]	10 [8, 13]	10 [7, 13]	<0.001
AGI score (%)						<0.001
AGI I	1813 (71.0)	1478 (74.5)	74 (66.7)	57 (65.5)	204 (55.3)	
AGI II	505 (19.8)	358 (18.0)	26 (23.4)	23 (26.4)	98 (26.6)	
AGI III	171 (6.7)	112 (5.6)	6 (5.4)	5 (5.7)	48 (13.0)	
AGI IV	63 (2.5)	37 (1.9)	5 (4.5)	2 (2.3)	19 (5.1)	
mNUTRIC score	5 [3, 6]	4 [3, 6]	6 [4, 7]	6 [5, 7.5]	6 [4, 7]	<0.001
Admission diagnosis						<0.001
Respiratory (%)	705 (27.4)	573 (28.9)	35 (31.5)	15 (17.2)	82 (22.2)	
Cardiovascular (%)	635 (24.9)	395 (19.9)	36 (42.4)	40 (46.0)	164 (44.4)	
CNS (%)	583 (22.8)	515 (25.9)	18 (16.2)	10 (11.5)	40 (10.8)	
Trauma (%)	320 (12.5)	285 (14.4)	12 (10.8)	7 (8.0)	16 (4.3)	
Gastrointestinal (%)	96 (3.8)	59 (3.0)	4 (3.6)	3 (3.4)	30 (8.1)	
Burn (%)	10 (0.4)	8 (0.4)	0	0	2 (0.5)	
Others (%)	203 (8.0)	150 (7.6)	6 (5.4)	12 (13.8)	35 (9.5)	
Post-operation (%)	482 (18.9)	410 (20.7)	15 (13.5)	11 (12.6)	46 (12.5)	<0.001
Comorbidity						
Hypertension (%)	1100 (43.1)	856 (43.1)	57 (51.4)	47 (54.0)	140 (37.9)	0.010
Diabetes (%)	445 (17.4)	307 (15.5)	28 (25.2)	23 (26.4)	87 (23.6)	<0.001
Coronary disease (%)	420 (16.5)	314 (15.8)	28 (25.2)	17 (19.5)	61 (16.5)	0.060
Stroke (%)	360 (14.1)	304 (15.3)	17 (15.3)	14 (16.1)	25 (6.8)	<0.001
Clinical outcomes						
28-day mortality (%)	379 (14.9)	277 (14.0)	22 (19.8)	21 (24.1)	59 (16.0)	0.021
28-day ICU-free days	7 [0, 17]	7 [0, 17]	5 [0, 17.5]	0 [0, 13.5]	7 [0, 17]	0.19
Organ support therapy-free days within the first 10 days after enrollment						
RT-free days	10 [10, 10]	10 [10, 10]	10 [10, 10]	10 [3.5, 10]	4 [0, 7]	<0.001
MV-free days	3 [0, 7]	0 [0, 6]	0 [0, 6]	3 [0, 6]	3 [0, 10]	0.004
VA-free days	10 [8, 10]	10 [8, 10]	10 [6.5, 10]	10 [8.5, 10]	10 [7, 10]	0.158

Data are presented as n (%) or median [first quartile, third quartile]. *BMI* Body mass index, *APACHE II* Acute Physiology and Chronic Health Evaluation II, *SOFA* Sequential Organ Failure Assessment, *mNUTRIC* modified Nutrition Risk in the Critically Ill, *AGI* acute gastrointestinal injury, *CNS* central nervous system, *RT* renal replacement therapy, *MV* mechanical ventilation, *VA* vasoactive agents, *AKI* acute kidney injury

### Nutrition therapy

**Figure 1:**
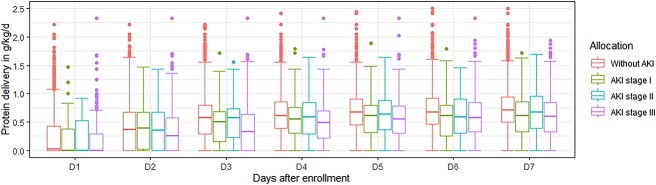
Daily protein intake from days 1 to 7 after enrollment. *AKI* acute kidney injury

Data regarding nutrition therapy is shown in Table 2. The mean time to start nutrition therapy was 1.93 days from study enrollment. EN accounted for the majority of nutrition delivery routes (applied in 2138/2552, 83.8% of patients). There was no significance in prokinetics use among patients with different grades of renal injury. Overall, between days 3 and 5 after enrollment, the mean protein delivery was 0.60 ± 0.38 g/kg/day and the mean energy delivery was 16.27 ± 9.45 kcal/kg/day. Feeding intolerance was frequent, as abdominal distension/pain, nausea/vomiting and diarrhea were present among 24.2, 3.0 and 12.5% of all patients, respectively. The daily protein intake from trial days 1–7 is shown in Figure 1. Patients with AKI generally had a lower protein intake than those without AKI (*p* < 0.001).

**Table 2:** Nutrition therapy in the study population among four different categories of renal function

	**Total**	**Without AKI**	**AKI stage I**	**AKI stage II**	**AKI stage III**	***P*** **value**
Number (%)	2552	1985 (77.8)	111 (4.3)	87 (3.4)	369 (14.5)	
Timing of nutrition therapy, day	1.93 (1.35)	1.87 (1.29)	2.01 (1.50)	1.95 (1.24)	2.25 (1.58)	<0.001
Initiation of EN within 48 h (%)	1478 (57.9)	1222 (61.6)	60 (54.1)	45 (51.7)	151 (40.9)	<0.001
Nutrition progress from 3–5 days after enrolment						
Length of nutrition support, day	2.60 (0.88)	2.64 (0.84)	2.48 (0.97)	2.59 (0.80)	2.38 (1.03)	<0.001
EN only (%)	1614 (63.2)	1291 (65.0)	66 (59.5)	53 (60.9)	204 (55.3)	0.003
PN only (%)	228 (8.9)	163 (8.2)	15 (13.5)	9 (10.3)	41 (11.1)	
EN + PN (%)	524 (20.5)	402 (20.3)	20 (18.0)	21 (24.1)	81 (22.0)	
None (%)	186 (7.3)	129 (6.5)	10 (9.0)	4 (4.6)	43 (11.7)	
Patients receiving prokinetics (%)	354 (14.0)	276 (14.0)	18 (16.2)	13 (14.9)	47 (12.8)	0.82
Mean protein and energy delivery from 3–5 days after enrolment						
Protein delivery, g/kg/day	0.60 (0.38)	0.63 (0.37)	0.55 (0.39)	0.59 (0.37)	0.49 (0.37)	<0.001
Energy delivery, kcal/kg/day	16.27 (9.45)	17.04 (9.43)	15.10 (9.25)	14.34 (8.60)	12.93 (8.98)	<0.001
Feeding intolerance (%)						
Abdominal distension/pain	617 (24.2)	449 (22.6)	26 (23.4)	21 (24.1)	121 (32.8)	0.001
Nausea/vomiting	76 (3.0)	55 (2.8)	6 (5.4)	3 (3.4)	12 (3.3)	0.437
Diarrhea	318 (12.5)	227 (11.4)	11 (9.9)	23 (26.4)	57 (15.4)	<0.001

Data are presented as n (%) or median [first quartile, third quartile] or mean (standard deviation). *AKI* Acute kidney injury, *EN* enteral nutrition, *PN* parenteral nutrition

### Associations between protein delivery and 28-day mortality

According to the multi-correlation analysis, SOFA score, APACHE II score and energy delivery were removed from the adjusted models for their correlation with mNUTRIC score and protein delivery, respectively (Figure S2, see online supplementary material). For the overall study cohort, the hazards of death decreased by 5% (HR = 0.95; 95%CI 0.92–0.98, *p* < 0.001) with each 0.1 g/kg/day increase in early protein delivery, adjusted for the following covariates: baseline AKI stage, gender, age, BMI, mNUTRIC score, AGI score and study group allocation in the NEED trial (Table S1, see online supplementary material).

**Figure 2:**
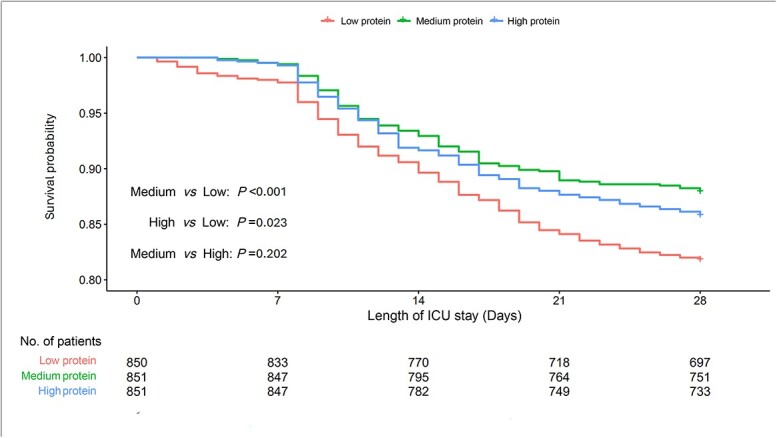
Kaplan–Meier survival curves for the association between different protein doses (divided by tertiles) and 28-day mortality. *ICU* intensive care unit

The results from the sensitivity analysis using mean protein doses from days 3 to 7 conform to the primary analysis (HR 0.94, 95%CI 0.91–0.97, *p* < 0.001; Table S2, see online supplementary material). Also, when stratifying the early protein delivery by tertiles, compared with low protein delivery, the risk of 28-day mortality decreased in the medium protein group (HR = 0.64; 95%CI 0.50–0.83, *p* < 0.001) and the high protein group (HR = 0.70; 95%CI 0.54–0.90, *p* = 0.006) after adjusting for the aforementioned confounders (Table S3, see online supplementary material). The Kaplan–Meier survival curves for the association between different protein groups and 28-day mortality are depicted in Figure 2.

### Interaction between early protein delivery and AKI stage

**Figure 3:**
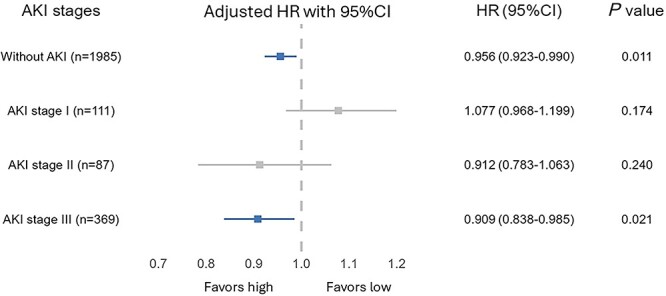
Associations between early protein delivery and 28-day mortality among patients with different AKI stages. *AKI* acute kidney injury, *HR* hazard ratio, *CI* confidence interval

In the subgroup multivariable analysis, the association between early protein delivery and 28-day mortality among patients with varying baseline AKI stage showed significant heterogeneity (interaction coefficient 0.93, test for interaction *p* = 0.028) (Table S4, see online supplementary material). The associations between early protein delivery, AKI stage and 28-day mortality are shown in Figure 3. Each 0.1 g/kg/day increase in early protein delivery was associated with a 4% reduction in 28-day mortality (HR = 0.96; 95%CI 0.92–0.99, *p* = 0.011) among patients without AKI at enrollment and 9% (HR = 0.91; 95%CI 0.84–0.99, *p* = 0.021) among patients with AKI stage III. However, no association between early protein delivery and mortality was detected in patients with AKI stage I (HR = 1.08; 95%CI 0.97–1.20, *p* = 0.174) and stage II (HR = 0.91; 95%CI 0.78–1.06, *p* = 0.240).

The adjusted HR values for 28-day mortality associated with early protein delivery in different AKI stage groups are depicted in Figure S3. Patients with AKI stages I and II were combined into one group, considering their relatively small sample sizes. The majority of the study patients received protein delivery in the range of 0–1.0 g/kg/day. Increasing the early protein delivery was associated with significantly decreasing mortality risk among patients without AKI and with AKI stage III, while no linear or non-linear pattern could be demonstrated among patients with AKI stages I–II.

## Discussion

This extensive *post hoc* analysis of the NEED trial showed that higher early protein delivery was associated with improved mortality up to 28 days in a cohort of critically ill patients with a mean protein delivery of 0.6 g/kg/day during early ICU stay (day 3–5). However, when considering baseline AKI stages, this association was only observed among patients without AKI or with AKI stage III rather than patients with AKI stages I and II.

There is controversy and a lack of rigorous data over how much protein should be given in the acute phase of critical illness. This uncertainty resulted in ambiguous recommendations in the current guidelines [4,5]. Recently, the FRANS observational study [27] demonstrated that >0.3 g/kg/day protein administration within 48 h of ICU admission was associated with worse outcomes. On the other hand, Zusman *et al*. [28] found that a higher mean protein intake was associated with improved mortality. However, these studies did not consider the potential impact of renal function, which is key in human protein metabolism [29].

When renal function is considered, in a post-analysis of the Nephro-Protective trial [11], higher protein showed mortality benefits for patients with normal renal function, but not in patients with AKI. Unfortunately, this was not replicated in the recent EFFORT-Protein trial [12], which showed no mortality benefits from higher protein provision (1.6 vs. 0.9 g/kg/day) in patients with normal renal function. Notably, it is challenging to compare the Nephro-Protective trial and the EFFORT-Protein trial as their routes of protein provision are different (PN vs. EN, respectively). Given that most ICU patients are fed enterally, the results of the Nephro-Protective trial may not be easily applicable. Our study filled the research gap about the potential effect of providing adequate protein (close to the guideline recommendation) during the late acute phase (from day 3 onward) on mortality in patients without AKI. In our study, higher protein delivery (up to 1.0 g/kg/day) was near the guideline recommendation since the mean protein delivery was 0.6 g/kg/day, as guidelines suggest progressively delivering proteins to the target. This result aligns with the observational PROTINVENT study [17] in which higher protein is associated with mortality benefits from day 4 onward but showed harm in the first three days. Taken together, providing protein nearer to the recommended amount during early ICU stay is associated with mortality benefits in patients without AKI, but higher protein intake may not bring additional benefits.

In patients with AKI, a recent *post hoc* analysis from the EFFORT-Protein trial showed that high protein (1.6 g/kg/day) may be associated with worse outcomes in all AKI stages [30], while our results suggest that protein intake close to the guideline’s recommendations confers clinical benefits in patients with AKI stage III, but not in patients with AKI stage I or II. This may be partly explained by the presence of RRT. In the former study, the adverse effects of high protein intake disappeared in patients receiving RRT, which is similar to our findings that close-to-recommendation protein intake was protective in AKI stage III, since 80.7% (298/369) of patients in the AKI stage III group underwent RRT on the first day after enrollment and 87.5% (323/369) of them received RRT during the first three days of enrollment.

The enhancement of mitochondrial biogenesis may explain the benefit of increased protein delivery. Using muscle biopsy, Carre *et al*. found that the early activation of mitochondrial biogenesis was associated with survival in ICU patients [31]. Branched amino acids improved mitochondrial biogenesis by targeting the mammalian site of the rapamycin (mTOR) pathway [32–35]. Moreover, mTOR is highly expressed in the kidney and regulates epithelial processes, which may explain the discrepancy between patients with or without AKI [36]. In patients with AKI, their ability to cope with the enhanced ureagenesis is compromised, leading to a decreased ability of organs to tolerate high levels of protein delivery and respond positively to high protein provision. When RRT is instituted, it alleviates the accumulation of urea and metabolites so that the beneficial effects of enhanced protein intake may not be counteracted or overwhelmed [37].

The results of this *post hoc* analysis should be interpreted cautiously, considering its strengths and limitations. The nutritional data in the research were prospectively collected during a multicenter randomised controlled trial with detailed records, implying good generalizability of the findings. Moreover, in this study, we defined early protein delivery as the mean protein doses between days 3 and 5 after enrollment, when early nutrition therapy (commenced within 48 h) was supposed to reach the feeding target [4,5]. However, as a *post hoc* analysis, residual confounding cannot be excluded for the non-randomised nature of the exposure, and we were unable to adjust the unrecorded confounders which may affect the regulation of metabolism and hyperinflammation, like the use of oxandrolone and beta blockers [38]. Also, the protein delivery in this study was generally lower than the recommended doses in the guidelines. It remains unclear whether an overall higher protein delivery would be associated with more significant differences in clinical outcomes. This study should be interpreted as hypothesis-generating, and no causal inferences can be made. However, it underlines the present guideline recommendations to deliver proteins in early critical illness progressively, and awaits more high-quality evidence to evaluate the role of renal function in protein delivery in critically ill patients, as well as to predict AKI at the early stage with several classic or novel biomarkers [39].

## Conclusions

This *post hoc* analysis showed that increased early protein delivery (up to close to guideline recommendations), as defined by days 3–5 after enrollment in the NEED trial, was associated with improved mortality in critically ill patients without AKI or with AKI stage III, when most of these patients received RRT, but not in those with AKI stages I and II.

## Abbreviations

AGI: Acute gastrointestinal injury; AKI: Acute kidney injury; APACHE II: Acute Physiology and Chronic Health Evaluation II; BMI: Body mass index; EN: Enteral nutrition; IQR: Interqurtile range; *mNUTRIC* Modified Nutrition Risk in the Critically Ill; PN: Parenteral nutrition; RRT: Renal replacement therapy; SOFA: Sequential Organ Failure Assessment. *mNUTRIC* modified Nutrition Risk in the Critically Ill.

## Supplementary Material

Supplement_Files_tkae027(1)

## References

[1] Fazzini B , Markl T, Costas C, Blobner M, Schaller SJ, Prowle J, et al. The rate and assessment of muscle wasting during critical illness: a systematic review and meta-analysis. Crit Care. 2023;27:2. 36597123 10.1186/s13054-022-04253-0 PMC9808763

[2] Wischmeyer PE . Tailoring nutrition therapy to illness and recovery. Crit Care. 2017;21:316. 29297385 10.1186/s13054-017-1906-8 PMC5751603

[3] Puthucheary ZA , Rawal J, McPhail M, Connolly B, Ratnayake G, Chan P, et al. Acute skeletal muscle wasting in critical illness. JAMA. 2013;310:1591–600. 24108501 10.1001/jama.2013.278481

[4] Singer P , Blaser AR, Berger MM, Calder PC, Casaer M, Hiesmayr M, et al. ESPEN practical and partially revised guideline: clinical nutrition in the intensive care unit. Clin Nutr. 2023;42:1671–89. 37517372 10.1016/j.clnu.2023.07.011

[5] Compher C , Bingham AL, McCall M, Patel J, Rice TW, Braunschweig C, et al. Guidelines for the provision of nutrition support therapy in the adult critically ill patient: the American Society for Parenteral and Enteral Nutrition. JPEN J Parenter Enteral Nutr. 2022;46:12–41. 34784064 10.1002/jpen.2267

[6] MacLaughlin HL , Friedman AN, Ikizler TA. Nutrition in kidney disease: Core curriculum 2022. Am J Kidney Dis. 2022;79:437–49. 34862042 10.1053/j.ajkd.2021.05.024

[7] Roberts PR , Black KW, Zaloga GP. Enteral feeding improves outcome and protects against glycerol-induced acute renal failure in the rat. Am J Respir Crit Care Med. 1997;156:1265–9. 9351632 10.1164/ajrccm.156.4.9607003

[8] Baylis C , Fredericks M, Wilson C, Munger K, Collins R. Renal vasodilatory response to intravenous glycine in the aging rat kidney. Am J Kidney Dis. 1990;15:244–51. 2305764 10.1016/s0272-6386(12)80769-4

[9] Doig GS , Simpson F, Bellomo R, Heighes PT, Sweetman EA, Chesher D, et al. Intravenous amino acid therapy for kidney function in critically ill patients: a randomized controlled trial. Intensive Care Med. 2015;41:1197–208. 25925203 10.1007/s00134-015-3827-9

[10] Allingstrup MJ , Kondrup J, Wiis J, Claudius C, Pedersen UG, Hein-Rasmussen R, et al. Early goal-directed nutrition versus standard of care in adult intensive care patients: the single-Centre, randomised, outcome assessor-blinded EAT-ICU trial. Intensive Care Med. 2017;43:1637–47. 28936712 10.1007/s00134-017-4880-3

[11] Zhu R , Allingstrup MJ, Perner A, Doig GS, Investigators N-PT, G. The effect of IV amino acid supplementation on mortality in ICU patients may Be dependent on kidney function: post hoc subgroup analyses of a Multicenter randomized trial. Crit Care Med. 2018;46:1293–301. 29771700 10.1097/CCM.0000000000003221

[12] Heyland DK , Patel J, Compher C, Rice TW, Bear DE, Lee ZY, et al. The effect of higher protein dosing in critically ill patients with high nutritional risk (EFFORT protein): an international, multicentre, pragmatic, registry-based randomised trial. Lancet. 2023;401:568–76. 36708732 10.1016/S0140-6736(22)02469-2

[13] Bufarah MNB , Costa NA, Losilla M, Reis NSC, Silva MZC, Balbi AL, et al. Low caloric and protein intake is associated with mortality in patients with acute kidney injury. Clinical nutrition ESPEN. 2018;24:66–70. 29576366 10.1016/j.clnesp.2018.01.012

[14] Kritmetapak K , Peerapornratana S, Srisawat N, Somlaw N, Lakananurak N, Dissayabutra T, et al. The impact of macro-and micronutrients on predicting outcomes of critically ill patients requiring continuous renal replacement therapy. PLoS One. 2016;11:e0156634. 27352307 10.1371/journal.pone.0156634 PMC4924859

[15] Berbel MN , Góes CR, Balbi AL, Ponce D. Nutritional parameters are associated with mortality in acute kidney injury. Clinics (Sao Paulo, Brazil). 2014;69:476–82. 25029579 10.6061/clinics/2014(07)06 PMC4081889

[16] Bellomo R , Cass A, Cole L, Finfer S, Gallagher M, Lee J, et al. Daily protein intake and patient outcomes in severe acute kidney injury: findings of the randomized evaluation of normal versus augmented level of replacement therapy (RENAL) trial. Blood Purif. 2014;37:325–34. 25171270 10.1159/000363175

[17] Koekkoek W , van Setten CHC, Olthof LE, Kars J, van Zanten ARH. Timing of PROTein INtake and clinical outcomes of adult critically ill patients on prolonged mechanical VENTilation: the PROTINVENT retrospective study. Clin Nutr. 2019;38:883–90. 29486907 10.1016/j.clnu.2018.02.012

[18] Ke L , Lin J, Doig GS, van Zanten ARH, Wang Y, Xing J, et al. Actively implementing an evidence-based feeding guideline for critically ill patients (NEED): a multicenter, cluster-randomized, controlled trial. Crit Care. 2022;26:46. 35172856 10.1186/s13054-022-03921-5 PMC8848648

[19] von Elm E , Altman DG, Egger M, Pocock SJ, Gotzsche PC, Vandenbroucke JP, et al. The strengthening the reporting of observational studies in epidemiology (STROBE) statement: guidelines for reporting observational studies. Int J Surg. 2014;12:1495–9. 25046131 10.1016/j.ijsu.2014.07.013

[20] Mehta RL , Kellum JA, Shah SV, Molitoris BA, Ronco C, Warnock DG, et al. Acute kidney injury network: report of an initiative to improve outcomes in acute kidney injury. Crit Care. 2007;11:R31. 17331245 10.1186/cc5713 PMC2206446

[21] Reintam Blaser A , Malbrain ML, Starkopf J, Fruhwald S, Jakob SM, De Waele J, et al. Gastrointestinal function in intensive care patients: terminology, definitions and management. Recommendations of the ESICM working group on abdominal problems. Intensive Care Med. 2012;38:384–94. 22310869 10.1007/s00134-011-2459-y PMC3286505

[22] Rahman A , Hasan RM, Agarwala R, Martin C, Day AG, Heyland DK. Identifying critically-ill patients who will benefit most from nutritional therapy: further validation of the ``modified NUTRIC'' nutritional risk assessment tool. Clin Nutr. 2016;35:158–62. 25698099 10.1016/j.clnu.2015.01.015

[23] Knaus WA , Draper EA, Wagner DP, Zimmerman JE. APACHE II: a severity of disease classification system. Crit Care Med. 1985;13:818–29. 3928249

[24] Vincent JL , Moreno R, Takala J, Willatts S, De Mendonca A, Bruining H, et al. The SOFA (sepsis-related organ failure assessment) score to describe organ dysfunction/failure. On behalf of the working group on sepsis-related problems of the European Society of Intensive Care Medicine. Intensive Care Med. 1996;22:707–10. 8844239 10.1007/BF01709751

[25] Lin J , Liu Y, Ke L, Li G, Lv C, Zhou J, et al. Feeding intolerance score in critically ill patients with enteral nutrition: a post hoc analysis of a prospective study. Nutr Clin Pract. 2022;37:869–77. 34679200 10.1002/ncp.10788

[26] F, H. Regression modeling strategies: with applications to linear models, logistic and ordinal regression, and survival analysis. Springer, 2015.

[27] Pardo E , Lescot T, Preiser JC, Massanet P, Pons A, Jaber S, et al. Association between early nutrition support and 28-day mortality in critically ill patients: the FRANS prospective nutrition cohort study. Crit Care. 2023;27:7. 36611211 10.1186/s13054-022-04298-1 PMC9826592

[28] Zusman O , Theilla M, Cohen J, Kagan I, Bendavid I, Singer P. Resting energy expenditure, calorie and protein consumption in critically ill patients: a retrospective cohort study. Crit Care. 2016;20:367. 27832823 10.1186/s13054-016-1538-4 PMC5105237

[29] Garibotto G , Sofia A, Saffioti S, Bonanni A, Mannucci I, Verzola D. Amino acid and protein metabolism in the human kidney and in patients with chronic kidney disease. Clin Nutr. 2010;29:424–33. 20207454 10.1016/j.clnu.2010.02.005

[30] Stoppe C , Patel JJ, Zarbock A, Lee ZY, Rice TW, Mafrici B, et al. The impact of higher protein dosing on outcomes in critically ill patients with acute kidney injury: a post hoc analysis of the EFFORT protein trial. Crit Care. 2023;27:399. 37853490 10.1186/s13054-023-04663-8 PMC10585921

[31] Carré JE , Orban JC, Re L, Felsmann K, Iffert W, Bauer M, et al. Survival in critical illness is associated with early activation of mitochondrial biogenesis. Am J Respir Crit Care Med. 2010;182:745–51. 20538956 10.1164/rccm.201003-0326OC PMC2949402

[32] Morio A , Tsutsumi R, Kondo T, Miyoshi H, Kato T, Narasaki S, et al. Leucine induces cardioprotection in vitro by promoting mitochondrial function via mTOR and Opa-1 signaling. Nutrition, metabolism, and cardiovascular diseases : NMCD. 2021;31:2979–86. 34362635 10.1016/j.numecd.2021.06.025

[33] Zhang L , Han J. Branched-chain amino acid transaminase 1 (BCAT1) promotes the growth of breast cancer cells through improving mTOR-mediated mitochondrial biogenesis and function. Biochem Biophys Res Commun. 2017;486:224–31. 28235484 10.1016/j.bbrc.2017.02.101

[34] Weichhart T . Mammalian target of rapamycin: a signaling kinase for every aspect of cellular life. Methods in molecular biology (Clifton, NJ). 2012;821:1–14. 10.1007/978-1-61779-430-8_1 22125056

[35] Romani M , Berger MM, D'Amelio P. From the bench to the bedside: branched amino acid and micronutrient strategies to improve mitochondrial dysfunction leading to sarcopenia. Nutrients. 2022;14:483. 10.3390/nu14030483. PMC8838610 35276842

[36] Grahammer F , Wanner N, Huber TB. mTOR controls kidney epithelia in health and disease. Nephrol Dial Transplant. 2014;29:i9–18. 24493874 10.1093/ndt/gft491

[37] Wooley JA , Btaiche IF, Good KL. Metabolic and nutritional aspects of acute renal failure in critically ill patients requiring continuous renal replacement therapy. Nutr Clin Pract. 2005;20:176–91. 16207655 10.1177/0115426505020002176

[38] Hundeshagen G , Blears E, Mertin V, Day AG, Palackic A, Tapking C, et al. Administration and effects of beta blockers and oxandrolone in severely burned adults: a post hoc analysis of the RE-ENERGIZE trial. Burns Trauma. 2024;12:tkad063. 10.1093/burnst/tkad063 PMC11033841 38650969

[39] Wang B , Xu J, Fu P, Ma L. MicroRNAs in septic acute kidney injury. Burns Trauma. 2023;11:tkad008. 36959845 10.1093/burnst/tkad008 PMC10027606

