# Supplementary material for: Early protein delivery in critically ill patients with acute kidney injury: post hoc analysis of a multicenter cluster-randomized controlled trial
Source: Burns Trauma. 2024 Jul 24;12:tkae027. doi: 10.1093/burnst/tkae027 (PMC11267585; doi:10.1093/burnst/tkae027)
Supplement: Supplement_Files_tkae027(1) [file supplement_files_tkae027(1).docx]

Supplement files

[Supplement Figure 1. Patients flowchart 2](#_Toc18607)

[Supplement Figure 2. Multi-correlation analysis for 28-day mortality 3](#_Toc3095)

[Supplement Figure 3. The trends of 28-day mortality with early protein delivery in different AKI stages 4](#_Toc8472)

[Supplement Table 1. Multivariable COX regression analysis of 28-day mortality 5](#_Toc19355)

[Supplement Table 2. Multivariable COX regression analysis of mean protein delivery from day 3-7 upon enrolment on 28-day mortality 6](#_Toc14827)

[Supplement Table 3. Multivariable Cox regression analysis of 28-day mortality by dividing the early protein delivery into three groups by tertiles 7](#_Toc26146)

[Supplement Table 4. Multivariable COX regression analysis of 28-day mortality considering the interaction between AKI stages and early protein delivery 8](#_Toc31224)

Figure S1. Patients flowchart


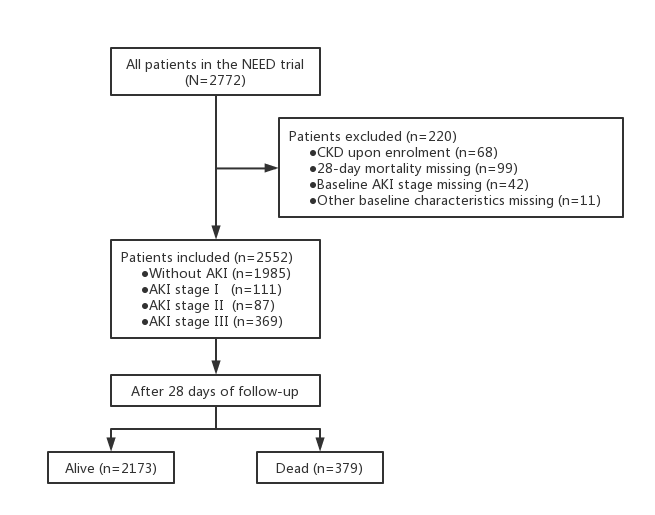


Figure S2. Multi-correlation analysis for 28-day mortality


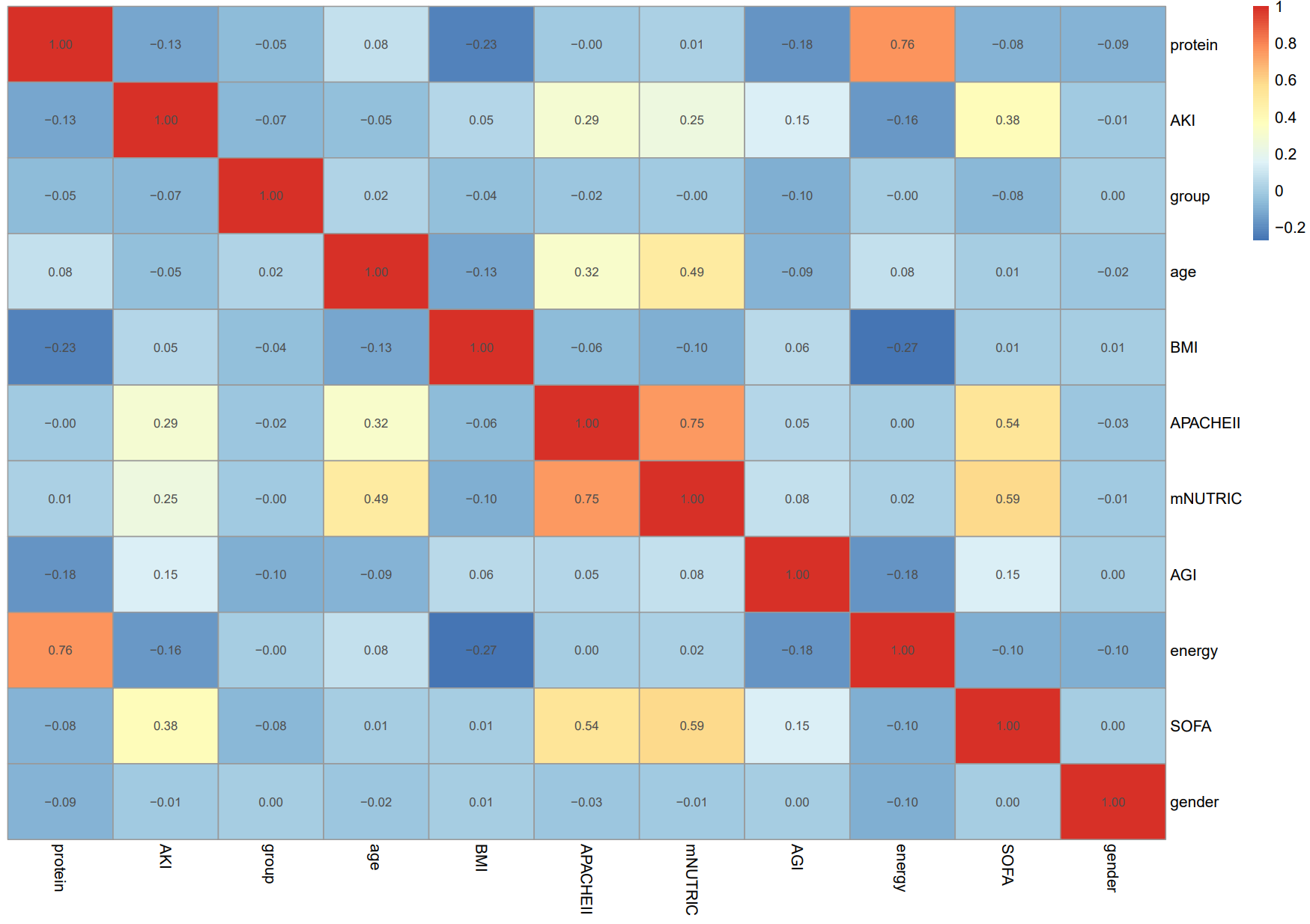


Figure S3. The trends of 28-day mortality with early protein delivery in different AKI stages


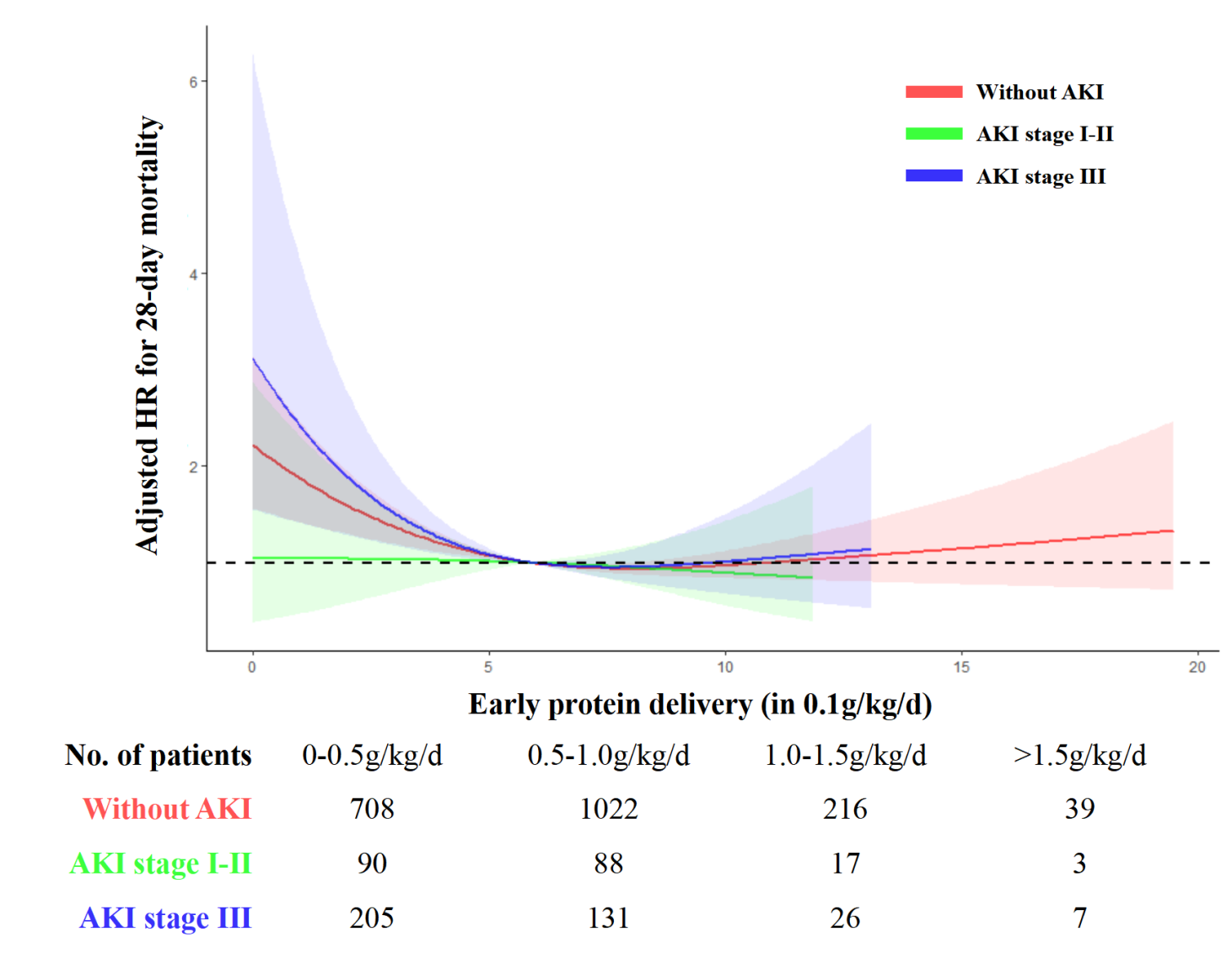


Table S1. Multivariable COX regression analysis of 28-day mortality

| **Variable** | **HR** | **95%CI** | **P-value** |
| --- | --- | --- | --- |
| Early protein delivery (in 0.1g/kg/d) | 0.95 | 0.92-0.98 | <0.001 |
| AKI stage | 0.98 | 0.77-1.24 | 0.859 |
| Gender (male) | 1.00 | 0.80-1.24 | 0.980 |
| Age | 1.01 | 1.00-1.01 | 0.050 |
| BMI | 0.93 | 0.83-0.96 | <0.001 |
| mNUTRIC score | 1.15 | 1.09-1.22 | <0.001 |
| AGI score | 0.99 | 0.62-1.57 | 0.965 |
| Group (intervention) | 0.93 | 0.76-1.14 | 0.489 |

*AKI* acute kidney injury, *BMI* Body Mass Index, *mNUTRIC* modified Nutrition Risk in the Critically ill, *AGI* Acute Gastrointestinal Injury

Table S2. Multivariable COX regression analysis of mean protein delivery from day 3-7 upon enrolment on 28-day mortality

| **Variable** | **HR** | **95%CI** | **P-value** |
| --- | --- | --- | --- |
| Mean protein delivery from day 3-7  (in 0.1g/kg/d) | 0.94 | 0.91-0.97 | <0.001 |
| AKI stage | 0.98 | 0.77-1.14 | 0.853 |
| Gender (male) | 0.99 | 0.80-1.23 | 0.957 |
| Age | 1.00 | 1.00-1.01 | 0.050 |
| BMI | 0.92 | 0.89-0.96 | <0.001 |
| mNUTRIC score | 1.16 | 1.09-1.22 | <0.001 |
| AGI score | 0.99 | 0.62-1.57 | 0.958 |
| Group (intervention) | 0.93 | 0.76-1.14 | 0.491 |

*AKI* acute kidney injury, *BMI* Body Mass Index, *mNUTRIC* modified Nutrition Risk in the Critically ill, *AGI* Acute Gastrointestinal Injury

Table S3. Multivariable Cox regression analysis of 28-day mortality by dividing the early protein delivery into three groups by tertiles

|  | **Low protein** | **Medium protein** | | | **High protein** | | |
| --- | --- | --- | --- | --- | --- | --- | --- |
|  |  | **HR** | **95%CI** | **P-value** | **HR** | **95%CI** | **P-value** |
| Early protein delivery | Reference | 0.64 | 0.50-0.83 | <0.001 | 0.70 | 0.54-0.90 | 0.006 |
| AKI stage | Reference | 1.12 | 0.85-1.48 | 0.436 | 0.98 | 0.74-1.29 | 0.870 |
| Gender (man) | Reference | 1.04 | 0.79-1.36 | 0.790 | 0.98 | 0.76-1.26 | 0.873 |
| Age | Reference | 1.00 | 1.00-1.01 | 0.271 | 1.01 | 1.00-1.02 | 0.068 |
| BMI | Reference | 0.91 | 0.87-0.95 | <0.001 | 0.95 | 0.91-0.98 | 0.006 |
| mNUTRIC score | Reference | 1.13 | 1.05-1.21 | <0.001 | 1.16 | 1.09-1.24 | <0.001 |
| AGI score | Reference | 1.00 | 0.59-1.69 | 0.995 | 1.04 | 0.64-1.71 | 0.862 |
| Group (intervention) | Reference | 1.22 | 0.95-1.57 | 0.124 | 0.88 | 0.69-1.27 | 0.319 |

*AKI* acute kidney injury, *BMI* Body Mass Index, *mNUTRIC* modified Nutrition Risk in the Critically ill, *AGI* Acute Gastrointestinal Injury

Table S4. Multivariable COX regression analysis of 28-day mortality considering the interaction between AKI stages and early protein delivery

| **Variable** | **HR** | **95%CI** | **P-value** |
| --- | --- | --- | --- |
| Early protein delivery (in 0.1g/kg/d) | 0.95 | 0.91-0.99 | 0.026 |
| AKI stage | 1.48 | 0.96-2.17 | 0.075 |
| Gender (man) | 1.00 | 0.80-1.24 | 0.060 |
| Age | 1.01 | 1.00-1.01 | 0.043 |
| BMI | 0.93 | 0.89-0.96 | <0.001 |
| mNUTRIC score | 1.15 | 1.09-1.22 | <0.001 |
| AGI score | 0.96 | 0.61-1.53 | 0.860 |
| Group (intervention) | 0.92 | 0.75-1.13 | 0.424 |
| Interaction (early protein delivery×AKI stage) | 0.93 | 0.87-0.99 | 0.028 |

*AKI* acute kidney injury, *BMI* Body Mass Index, *mNUTRIC* modified Nutrition Risk in the Critically ill, *AGI* Acute Gastrointestinal Injury, *APACHE II* Acute Physiology and Chronic Health Evaluation II
